# Knee Injury and Osteoarthritis Outcome Score Trajectories After Primary Total Knee Arthroplasty in United States Veterans

**DOI:** 10.7759/cureus.36670

**Published:** 2023-03-25

**Authors:** Bryce W Polascik, Maggie Horn, Shreyas Pyati, Edward R Mariano, J.P. Ginsberg, Karthik Raghunathan

**Affiliations:** 1 Institute for Medical Research, Durham Veterans Affairs Health Care System, Durham, USA; 2 Department of Orthopaedic Surgery, Duke University School of Medicine, Durham, USA; 3 Department of Anesthesiology, Stanford University School of Medicine, Stanford, USA; 4 Anesthesiology and Perioperative Medicine, Palo Alto Veterans Affairs Health Care System, Palo Alto, USA; 5 Department of Anesthesiology, Duke University School of Medicine, Durham, USA

**Keywords:** outcome scores, tka, koos, knee osteoarthritis, primary total knee arthroplasty, knee arthroplasty, veterans

## Abstract

Introduction: The volume of total knee arthroplasty (TKA) procedures continues to increase, including among United States (US) veterans, but there is little data characterizing recovery using validated knee-related questionnaires.

Methods: In this prospective cohort study, we sought to establish the feasibility of longitudinal characterization of recovery after TKA using the validated Knee Injury and Osteoarthritis Outcome Score (KOOS), specifically focusing on two of the KOOS subscales (pain and quality of life (QOL)). We solicited participants who agreed to fill out these knee-related questionnaires preoperatively and 3, 6, and 12 months after discharge following unilateral TKA within the Durham Veterans Affairs Health Care System. We examined rates of prospective completion of the KOOS and face validity of scores at each study time point. We transformed and reported scores on the 0-100 scale, with zero representing significant knee pain or poor QOL and 100 representing no knee pain or good QOL.

Results: Of 200 US veterans presenting between May 2017 and 2018, 21 (10.5%) agreed to participate by filling out the KOOS questionnaire longitudinally from before surgery until one year after discharge. All 21 (100%) participants were male and completed the two KOOS subscale questions (pain and QOL) preoperatively. Of those, 16 (76.2%) also completed KOOS at 3 months, 16 (76.2%) at 6 months, and seven (33.3%) at 12 months. Compared to mean preoperative values (pain: 33.47 + 6.78, QOL: 11.91 + 4.99), the KOOS subscale scores had significantly improved by 6 months after TKA (pain: 74.41 + 10.72, QOL: 49.61 + 13.25) but plateaued at 12 months (pain: 74.60 + 20.80, QOL: 50.89 + 20.61). The magnitude of improvement in absolute scores, pain and QOL, was similar and significant at 12 months compared to preoperative values with an increase of 41.13 (p=0.007) and 38.98 (p=0.009), respectively.

Conclusion: Primary TKA in US veterans with advanced osteoarthritis may lead to improved patient-reported KOOS pain and QOL subscale measures at 12 months compared to preoperative scores, with the majority of improvement occurring by 6 months. Only one in ten US veterans approached preoperatively agreed to complete the validated knee-related outcomes questionnaire prior to undergoing TKA. About three-quarters of those veterans also completed it both three and six months after discharge. Collected KOOS subscale scores demonstrated face validity and showed substantial improvement in pain and QOL over the six-month postoperative period. Only one in three veterans who completed the KOOS questionnaire preoperatively also completed it at 12 months, but this does not support the feasibility of follow-up assessments beyond 6 months. To better understand longitudinal pain and QOL trajectories in US veterans undergoing primary TKA for advanced osteoarthritis and to improve study participation, additional research using the KOOS questionnaire may add further insights into this underreported population.

## Introduction

It has been demonstrated that total joint arthroplasty lessens pain and enhances the quality of life (QOL) in individuals with advanced osteoarthritis [1]. In particular, total knee arthroplasty (TKA) has been increasingly utilized to alleviate painful osteoarthritis impacting QOL [2]. As such, the number of primary TKA procedures conducted in the United States (US) across all healthcare systems is estimated to rise to almost 3.5 million annually by 2030 [3]. As an intervention for advanced, painful knee osteoarthritis, TKA also restores function [4], contributing to adaptation and advancement in perioperative care to further optimize postoperative outcomes [2,4] and improve QOL.

Outcomes and recovery trajectories following TKA, however, have not been fully explored in the US veteran patient population with painful osteoarthritis. Much-needed information on such trajectories in this cohort cannot be extrapolated from non-veteran patient populations due to known differences in sociodemographics, medical comorbidities, and health-related QOL scores between groups [5,6]. In addition, the US veteran population manifests increased numbers of medical comorbidities, including diabetes, obesity, cancer, and kidney disease, among others, which affect both function and pain trajectories following surgical intervention. Since the entry of veterans into the Veterans Affairs Health Care System (VAHCS) has been traditionally governed by specific eligibility criteria, only about 10% of all US veterans use VA health services [6], and it is this sub-population that is particularly unique. Thus, health-related studies performed specifically in veteran populations that receive care in the VAHCS are needed to better understand their outcomes.

The Knee Injury and Osteoarthritis Outcome Score (KOOS) is an accepted, validated, and reliable patient-reported outcome measure following TKA and has been used in registries and value-based payment models [7-10] to identify effective interventions [10]. Because the KOOS questionnaire can be administered in 10 minutes, used longitudinally, and has been validated in individuals aged 13-79 years, it can assess patient opinion about knee-associated morbidity and can be used to study recovery trajectories [10].

The management of perioperative pain following TKA has improved with increasing emphasis on multimodal analgesia to minimize opioid use [11]. Our anesthesiology and acute pain management group at the Durham VAHCS serially uses KOOS to measure postoperative outcomes using the KOOS pain and QOL subscales. We aimed to assess pain and QOL recovery trajectories over 12 months using KOOS in US veterans with advanced osteoarthritis undergoing TKA.

## Materials and methods

This prospective cohort study (#1968) was approved on October 21, 2016, by the Institutional Review Board at VAHCS in Durham, North Carolina, and conducted in compliance with the 1996 Health Insurance Portability and Accountability Act and adhered to all tenets of the Declaration of Helsinki. All participants provided written informed consent. This manuscript adheres to applicable EQUATOR guidelines. All data were de-identified.

Veterans with advanced knee osteoarthritis who were undergoing TKA at the Durham VAHCS between May 2017 and May 2018 were eligible. The TKA procedure was performed at the Durham VAHCS by orthopedic surgeons who were employed at both the Durham VAHCS and its academic affiliate, the Duke University Health System. Inclusion criteria included completion of the self-administered KOOS questionnaire and the administration of the standard multimodal analgesic protocol for acute perioperative pain management. The standard multimodal analgesic protocol included preoperative oral acetaminophen 975 mg and celecoxib 400 mg, intraoperative spinal anesthesia with hyperbaric bupivacaine 10-15 mg, intraoperative or postoperative placement of a continuous nerve block catheter infusing 0.2% ropivacaine at 6-8 cc/hour beginning after the spinal anesthetic had worn off and up to 72 hours postoperatively, postoperative oral opioid oxycodone immediate release 5-10 mg every six hours as needed as well as intravenous hydromorphone 0.25-0.5 mg every two or three hours as needed, and postoperative scheduled nonopioids oral meloxicam 5-10 mg daily and oral acetaminophen 650 mg every six hours. Discharge prescriptions varied based on patient analgesic needs and included oral oxycodone-acetaminophen immediate release 5-325 mg every six hours as needed and oral meloxicam 5-10 mg daily for 10 days. Further quantitative details were not collected.

All KOOS questions were completed by the patient on paper and answered based on their experience during the previous week. The KOOS questionnaire consists of 42 questions that comprise five validated subscales: pain, symptoms, activities of daily living (ADL) function, sport and recreation function, and QOL [10]. A Likert scale was used, and each KOOS question within each subscale has five response options, from zero (none/never/not at all) to four (always/extreme/constantly/totally/extremely). A total KOOS score for all five subscales was not calculated. Two of the KOOS subscales, pain and QOL, formed the principal outcome measures in our study. The pain subscale consists of nine items to gauge knee pain, including frequency as well as amount while twisting/pivoting, straightening, bending fully, walking on a flat surface, going up or down stairs, while in bed, sitting/lying, and standing upright [10]. The QOL subscale consists of four items to assess QOL, including awareness of knee problems, lifestyle modifications, lack of confidence, and general difficulty with the knee [10]. Each subscale score was calculated as the sum of each of the questions within that subscale. At each study time point, mean scores were then separately calculated for each subscale and were transformed to a 0-100 scale, with zero representing significant knee pain or poor QOL and 100 representing no knee pain or good QOL, for statistical analysis [10,12]. We also examined rates of prospective completion of the KOOS and face validity of scores at each study time point.

The trajectories of KOOS pain and QOL subscale measures across 12 months were assessed. The primary outcomes were the change in the mean score of each KOOS pain and QOL subscale domain from preoperatively to 12 months postoperatively. These two KOOS subscale scores at the three-month and six-month visits were secondary outcomes.

Specific patient characteristics, duration and type of non-surgical treatments, and pain management patterns were not available. Mean KOOS pain scores and QOL scores as well as margins of error were calculated at each time point. Unpaired t-tests compared each of the KOOS subscale mean differences at each of the four time points. A two-sided p<0.05 was considered significant. The absolute change score with a margin of error was calculated for the preoperative to 12-month interval, preoperative to 3-month interval, 3-month to 6-month interval, and 6-month to 12-month interval. Confidence intervals (95%) were also calculated for each change score.

## Results

At the Durham VAHCS, 200 eligible US veterans with advanced osteoarthritis undergoing unilateral primary TKA were approached during the study period, and 21 (10.5%) agreed to participate. All 21 (100%) were male and underwent unilateral primary TKA as scheduled. All 21 veterans received the standard multimodal analgesic protocol for acute perioperative pain management as described. All 21 (100%) completed the KOOS questionnaire preoperatively; 16 (76.2%) completed KOOS at 3 and 6 months, and 7 (33.3%) completed KOOS at the 12-month study timepoint.

**Figure 1:**
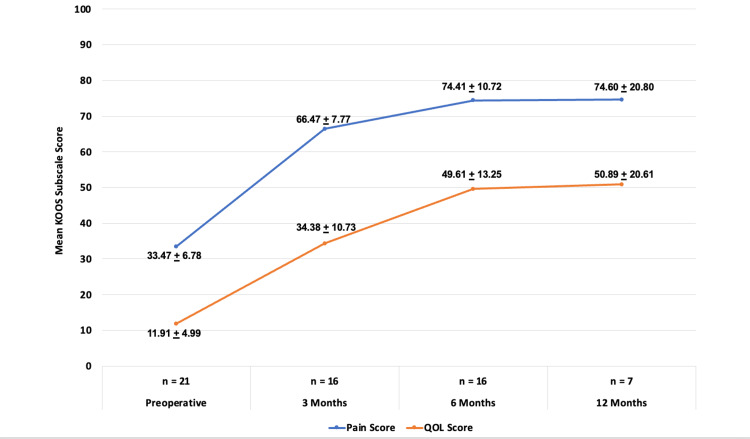
Mean KOOS pain and QOL subscale scores after primary TKA in US veterans over 12 months KOOS: knee injury and osteoarthritis outcome score. QOL: quality of life

Compared to preoperative values (pain: 33.47 + 6.78, QOL: 11.91 + 4.99), mean KOOS pain and QOL scores improved in US veterans after primary TKA during the 12-month postoperative period (Figure 1).

**Figure 2:**
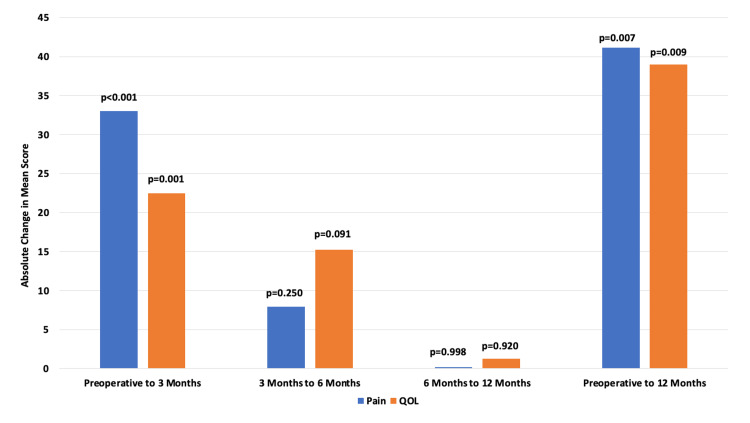
Absolute change scores for KOOS pain and QOL subscales following primary TKA in US veterans. For mean pain score, 95% CI was 22.70 to 43.32 at 3 months, -5.29 to 21.18 at 6 months, and -23.21 to 23.59 at 12 months. For mean QOL score, 95% CI was 10.64 to 34.30 at 3 months, -1.82 to 32.28 at 6 months, and -23.22 to 25.79 at 12 months. Over 12 months, 95% CI was 19.26 to 63.01 for mean pain score and 17.78 to 60.18 for mean QOL score. QOL: quality of life

Over 12 months, KOOS pain scores improved from a mean of 33.47 + 6.78 preoperatively to 74.60 + 20.80 at 12 months, for a total change score of 41.13 + 21.88 (95% CI, 19.26 to 63.01) (p=0.007). Both scores peaked at 6 months (pain: 74.41 + 10.72, QOL: 49.61 + 13.25) and were then maintained through 12 months (pain: 74.60 + 20.80, QOL: 50.89 + 20.61) (Figure 1). At three months postoperatively, KOOS pain scores improved from a mean of 33.47 + 6.78 preoperatively to 66.47 + 7.77, for a change score of 33.00 + 10.31 (95% confidence interval (CI), 22.70 to 43.32) (p<0.001) (Figure 2).

At six months, KOOS pain scores improved slightly from a mean of 66.47 + 7.77 at three months to 74.41 + 10.72, for a change score of 7.94 + 13.24 (95% CI, -5.29 to 21.18) (p=0.250) (Figure 2). At 12 months, KOOS pain scores were maintained, with a mean of 74.41 + 10.72 at 6 months and 74.60 + 20.80 at 12 months, for a change score of only 0.19 + 23.40 (95% CI, -23.21 to 23.59) (p=0.988) (Figure 2).

Over 12 months, KOOS QOL scores improved from a mean of 11.91 + 4.99 preoperatively to 50.89 + 20.61 at 12 months, for a total change score of 38.98 + 21.20 (95% CI, 17.78 to 60.18) (p=0.009). Three months postoperatively, KOOS QOL scores improved from a mean of 11.91 + 4.99 preoperatively to 34.38 + 10.73, for a change score of 22.47 + 11.83 (95% CI, 10.64 to 34.30) (p=0.001) (Figure 2). At six months, QOL scores improved from a mean of 34.38 + 10.73 at three months to 49.61 + 13.25, for a change score of 15.23 + 17.05 (95% CI, -1.82 to 32.28) (p=0.091) (Figure 2). At 12 months, QOL scores were maintained, with a mean of 49.61 + 13.25 at 6 months and 50.89 + 20.61 at 12 months, for a change score of only 1.28 + 24.50 (95% CI, -23.22 to 25.79) (p=0.920) (Figure 2).

Over 12 months, the magnitude of improvement in mean pain and QOL scores from preoperative values was similar, with a change score of 41.13 + 21.88 and 38.98 + 21.20, respectively.

## Discussion

This prospective cohort study assessed US veterans undergoing TKA for advanced osteoarthritis who completed the KOOS questionnaire preoperatively as well as at 3, 6, and 12 months postoperatively. The serial assessment of two validated KOOS subscales, pain and QOL, estimated one-year recovery trajectories, demonstrating that both pain and QOL improved significantly after TKA in this V-veteran cohort. Most of the improvement in both subscales occurred within the first six months after surgical intervention. Pain scores appeared to improve more than QOL scores within the first three months postoperatively; however, between three and six months, QOL scores improved more than pain scores. As TKA continues to become increasingly widespread [3], outcomes data to better understand the procedure’s efficacy are needed in diverse patient populations, including US veterans. Self-reported KOOS pain and QOL scores can be measured longitudinally in nearly all patients who complete the questionnaire [9,13-15].

Our data are a meaningful addition to the existing body of research that examines veteran-centered TKA outcomes [15,16]. Singh and Sloan reported cross-sectional, health-related QOL data using short-form 36 in 531 US veterans undergoing primary TKA over 20 years ago and concluded that TKA may contribute to poorer physical but not mental QOL [16]. The KOOS QOL subscale, however, was not used. Frisch et al. assessed 30-day outcomes after primary TKA in US veterans compared to a civilian cohort but did not include KOOS. They found that the rate of having a greater than four-day length of stay, of being readmitted within 30 days, and of developing complications within 30 days postoperatively was higher in veterans [15]. Pain nor QOL were assessed.

In a randomized controlled trial of non-veterans in Denmark who completed 12 months of follow-up, Skou et al. administered the KOOS questionnaire to 46 Danish patients with moderate to severe knee osteoarthritis who underwent primary TKA followed by 12 weeks of nonsurgical treatment which included dietary advice, exercise, education, pain medication, and use of insoles, and 49 who were randomized to 12 weeks of nonsurgical treatment followed by TKA in 13 participants [14]. Preoperatively, each cohort had less pain and better QOL than the US veterans in our study. At 12 months, the TKA cohort had less pain and better QOL than the US veterans in our study. Our veterans had less pain and a similar QOL than the Danish nonsurgical cohort. Both Danish cohorts and our veteran cohort had less pain and better QOL as measured by KOOS 12 months postoperatively, with the nonsurgical cohort achieving the smallest improvement in both metrics [14]. The Danish cohort that underwent TKA achieved a similar improvement in QOL as US veterans who underwent TKA, but a smaller magnitude of pain relief than US veterans. These comparative findings may be a consequence of differences in nonsurgical treatments, social or cultural practices, and/or variable confounders including operative and analgesic differences. Future work will provide clarification.

This study has some limitations. The 21 veterans who participated may be highly motivated and likely to do well following TKA, and thus our findings may be attributed to sampling bias. Reasons for non-participation were not collected; however, further research will identify barriers to study participation and study completion. The presence of individual non-response bias during follow-up may limit generalizability. In addition, the study findings may not be generalizable to women veterans, civilians, or veterans undergoing primary TKA at other VAHCS facilities or non-VAHCS sites with different operative and analgesic practices. There are variables unaccounted for that may confound our findings. We did not have detailed quantitative information regarding perioperative opioids and non-opioids nor did we include other KOOS subscales from symptoms, ADL function, or sport and recreation function, which may affect conclusions regarding the efficacy of TKA and overall recovery trajectories in veterans.

## Conclusions

The findings in this pilot study suggest that primary TKA in USvVeterans with advanced osteoarthritis may lead to improved patient-reported KOOS pain and QOL subscale measures at 12 months compared to preoperative scores, with the majority of improvement occurring by 6 months. This pilot study also examined the feasibility of longitudinal assessment of knee-related pain and QOL after surgical intervention and found that only one in 10 US veterans who were approached preoperatively agreed to complete the validated knee-related outcomes questionnaire prior to undergoing TKA. About three-quarters of those veterans also completed it both three and six months after discharge. Collected KOOS subscale scores demonstrated face validity and showed substantial improvement in pain and QOL over the six-month postoperative period. Only one in three veterans who completed the KOOS questionnaire preoperatively also completed it at 12 months, however, which does not support the feasibility of follow-up assessments beyond six months. Routine longitudinal assessment of knee-related pain and QOL recovery trajectories after TKA seems feasible up to six months after discharge using the KOOS questionnaire. To better understand longitudinal pain and QOL trajectories in US veterans undergoing primary TKA for advanced osteoarthritis and to improve study participation, an additional study using the KOOS questionnaire may add further insights into this underreported population.
